# Asian consensus on normothermic intraperitoneal and systemic treatment for gastric cancer with peritoneal metastasis

**DOI:** 10.1007/s10120-025-01631-9

**Published:** 2025-07-14

**Authors:** Zhenggang Zhu, Joji Kitayama, Hyung-Ho Kim, Jimmy Bok-Yan So, Hui Cao, Lin Chen, Xiangdong Cheng, Jiankun Hu, Motohiro Imano, Hironori Ishigami, Ye Seob Jee, Jong-Han Kim, Yasuhiro Kodera, Han Liang, Xiaowen Liu, Sheng Lu, Yiping Mou, Mingming Nie, Won Jun Seo, Yanong Wang, Dan Wu, Zekuan Xu, Hironori Yamaguchi, Chao Yan, Zhongyin Yang, Kai Yin, Yutaka Yonemura, Wei-Peng Yong, Jiren Yu, Jun Zhang, Asian Gastric Cancer NIPS Treatment Collaborative Group

**Affiliations:** 1 Department of General Surgery, Shanghai Key Laboratory of Gastric Neoplasms, Ruijin Hospital, Shanghai Jiao Tong University School of Medicine, Shanghai Institute of Digestive Surgery, Shanghai, China; 2 Department of Gastrointestinal Surgery, Clinical Research Center, Jichi Medical University, Jichi Medical University Hospital, Shimotsuke, Japan; 3 Department of Surgery, Seoul National University Bundang Hospital, Seongnam, Korea; 4 Department of Surgery, Seoul National University College of Medicine, Seoul, Korea; 5 Department of Surgery, National University Hospital, Singapore, Singapore; 6 Department of Surgery, National University of Singapore, Singapore, Singapore; 7 Department of General Surgery, Renji Hospital, Shanghai Jiao Tong University School of Medicine, Shanghai, China; 8 Department of General Surgery, Peking University International Hospital, Beijing, China; 9 Zhejiang Provincial Research Center for Upper Gastrointestinal Tract Cancer, Zhejiang Cancer Hospital, Hangzhou, China; 10 Department of Gastrointestinal Surgery and Laboratory of Gastric Cancer, West China Hospital, Sichuan University, Chengdu, China; 11 Department of Surgery, Faculty of Medicine, Kindai University, Higashiosaka, Japan; 12 Department of Chemotherapy, The University of Tokyo Hospital, Bunkyo, Japan; 13 Department of Gastrointestinal Surgery, Graduate School of Medicine, The University of Tokyo, Bunkyo, Japan; 14 Department of Surgery, Dankook University Hospital, Dankook University College of Medicine, Cheonan, Korea; 15 Department of Surgery, Korea University Guro Hospital, Korea University College of Medicine, Seoul, Korea; 16 Department of Gastroenterological Surgery, Nagoya University Graduate School of Medicine, Nagoya, Japan; 17 Department of Gastric Cancer, Tianjin Medical University Cancer Institute & Hospital, Tianjin, China; 18 Department of Gastric Cancer Surgery, Fudan University Shanghai Cancer Center, Shanghai, China; 19 Department of Oncology, Shanghai Medical College, Fudan University, Shanghai, China; 20 Department of Gastroenterology & Pancreatic Surgery, Zhejiang Provincial People’s Hospital, Hangzhou, China; 21 Department of General Surgery, Changhai Hospital, Second Military Medical University, Shanghai, China; 22 Division of Foregut Surgery, Department of Surgery, Korea University College of Medicine, Seoul, Korea; 23 Department of General Surgery, The Second Affiliated Hospital of Medical College, Hangzhou, China; 24 Department of General Surgery, Jiangsu Province Hospital, First Affiliated Hospital of Nanjing Medical University, Nanjing, China; 25 Department of Surgery, Division of Clinical Oncology, Jichi Medical University, Shimotsuke, Japan; 26 Peritoneal Dissemination Center, Kishiwada Tokushukai Hospital, Kishiwada, Japan; 27 Department of Haematology-Oncology, National University Cancer Institute, Singapore, Singapore; 28 Department of Gastrointestinal Surgery, The First Affiliated Hospital, Zhejiang University School of Medicine, Hangzhou, China; 29 Department of Oncology, Shanghai Key Laboratory of Gastric Neoplasms, Ruijin Hospital, Shanghai Jiao Tong University School of Medicine, Shanghai Institute of Digestive Surgery, Shanghai, China

**Keywords:** Gastric cancer, Peritoneal metastasis, Normothermic intraperitoneal chemotherapy, NIPS, Conversion surgery, Consensus

## Abstract

Peritoneal metastasis (PM) is a major challenge in advanced gastric cancer (GC) with poor prognosis. Normothermic intraperitoneal and systemic treatment (NIPS) has become a promising therapeutic approach. This consensus aims to provide practical recommendations for NIPS treatment for gastric cancer with peritoneal metastasis (GCPM). The GRADE standards were used to rank evidence, and the Delphi method was employed for expert voting. 30 experts from China, Japan, South Korea, and Singapore participated in the development of this consensus. 28 experts participated in the voting process, which produced 29 statements covering diagnostic approaches, patient selection criteria, treatment regimens, management of intraperitoneal port placement, and conversion surgery considerations, and post-surgical treatment strategies in NIPS therapy. Based on current evidence and expert experience, these statements aim to improve the clinical outcomes of NIPS therapy for GCPM patients.

## Introduction

Gastric cancer (GC) is a prevalent malignant gastrointestinal tumor, ranking fifth in incidence and third in mortality globally [1]. In 2020, over half a million individuals were diagnosed with peritoneal metastasis (PM) from GC [2]. The peritoneum is one of the most common sites of recurrence and metastasis in advanced gastric cancer (AGC) worldwide, especially for the scirrhous type with serosal exposure [3–5]. Approximately 20% of patients are diagnosed with PM before or during surgery, and a significant portion of patients with T3 and T4 stage develop PM after radical surgery [6]. Historically, PM was considered incurable, with aggressive treatment often not recommended. The median survival time for PM patients is only 5–11 months, and the 5 year survival rate is less than 2% [7, 8].

With advancements in understanding the pathology of PM, it can sometimes take the form of a regional disease, with macroscopic lesions confined to the peritoneal surface. Recently, a novel approach called normothermic intraperitoneal and systemic chemotherapy (NIPS) has emerged [9]. This comprehensive treatment has shown potential improvements in survival rates for PM patients [10–14]. Some patients have even achieved clinical cure, a prospect previously thought impossible. Thus, NIPS is introduced in pursuit of effective PM treatment options.

This consensus aims to standardize the NIPS treatment approach for gastric cancer with peritoneal metastasis (GCPM). The essential aspects include the diagnosis and assessment of PM, criteria for laparoscopic exploration and NIPS treatment, treatment regimen, prevention of complications, criteria for conversion surgery, etc. The GRADE collaboration standards were used to rank evidence, and the Delphi method was employed for expert voting and statements revisions. To ensure the comprehensiveness of this consensus, 30 experts from related fields across different countries, including China, Japan, South Korea, and Singapore, were invited for consultation, review, and modification of the statements. Among these experts, 28 participated in the voting process. Voting results are categorized into six levels: 1. complete agreement (100%); 2. basic agreement (80%); 3. partial agreement (60%); 4. partial disagreement (40%); 5. more disagreement (20%); and 6. complete disagreement (0). This process produced 29 statements. Statements that receive over 80% "Complete agreement" votes are given a strong recommendation. If a statement receives over 80% combined "Complete agreement" and "Basic agreement" votes, it gets a weak recommendation.

## The statement of NIPS

Statement 1: Define NIPS as normothermic intraperitoneal and systemic treatment.

(Quality of Evidence: D Level of Recommendation: Weak; Degree of Expert Agreement: 85.7%).

Combination of systemic and intraperitoneal chemotherapy (SIPC) was proposed and began to be applied in the 1990s, but failed to provide significant clinical benefits possible due to the lack of pharmacological advantages. In contrast, SIPC using Taxane demonstrated the promising results in multiple Phase II trials [12–14]. As conversion therapy developed, neoadjuvant intraperitoneal and systemic chemotherapy (NIPS) was introduced in 2006 [9]. While neoadjuvant chemotherapy typically implies subsequent surgery, not all GCPM patients can undergo surgery post-chemotherapy. To address this, bidirectional intraperitoneal and systemic induction chemotherapy (BISIC) was introduced in 2014 [15]. In addition, the term normothermic intraperitoneal chemotherapy (NIPEC) has been used to differentiate it from hyperthermic intraperitoneal chemotherapy (HIPEC) [16].

Given the prevalence of the term NIPS and its common association with systemic treatment, we propose to redefine NIPS as "normothermic intraperitoneal and systemic treatment" (NIPS) for the following reasons:

- For GCPM patients, intraperitoneal combined with systemic treatment does not always lead to surgery, making "neoadjuvant" inappropriate.
- NIPS can be applied not only preoperatively but also for patients with postoperative PM recurrence, and even prophylactically for those at high risk of PM recurrence.
- With the advancement of immunotherapy and targeted therapy, systemic treatment is no longer limited to chemotherapy, thus "chemotherapy" should be updated to "treatment".
- NIPS significantly differs from HIPEC (which is usually administered intraoperatively during surgery), and "normothermic" highlights this distinction.
- The term NIPS has relatively high acceptance.

## Overview of PM from gastric cancer

Statement 2: GCPM is the spread of cancer cells from the stomach to the peritoneum.

(Quality of Evidence: C; Level of Recommendation: Weak; Degree of Expert Agreement: 100%).

PM is the most common route for AGC spread and the leading cause of death, even after curative resection [17]. Major findings include [18]:

- Microscopic PM is present in about 10–20% of patients with T3 or T4 GC undergoing radical surgery.
- PM is the initial and exclusive site of recurrence in approximately 40–60% of AGC patients after resection with curative intent.
- PM directly causes death in about 20–40% of GC patients.
- Younger GC patients, especially young women, are prone to PM compared to older patients.

The exact mechanism of GCPM is not fully understood. The widely accepted "seed and soil" hypothesis suggests PM involves several stages [19, 20]:

- Cancer cells penetrate the serous membrane and shed into the abdominal cavity.
- These cells adhere to specific areas of the peritoneum.
- Cancer cells invade local tissue, induce neovascularization, and develop into metastatic foci.

Some researchers also propose that PM might occur via hematogenous and lymphatic pathways [21, 22]. Advances in genomic sequencing have provided deeper insights into the biology of PM, revealing novel therapeutic targets and suggesting the need for peritoneal-directed treatment strategies [23].

Statement 3: Free cancer cells (FCCs) in the peritoneal cavity are a prerequisite for PM. FCCs (P0CY1) serve as an independent marker for stage IV GC.

(Quality of Evidence: A; Level of Recommendation: Strong; Degree of Expert Agreement: 92.9%).

Identifying FCCs in the abdominal cavity is crucial for early detection of PM. If FCCs are positive, even without other incurable factors, the 5-year survival rate with chemotherapy after surgery is about 25–28%[24, 25]. Since 1998, the Japanese Gastric Cancer Association (JGCA) has recommended peritoneal lavage cytology (PLC) to detect FCCs in GC. In 2010, the American Joint Committee on Cancer (AJCC) included FCCs positivity (CY1) as a criterion for distant metastasis (M1) in its 7th edition TNM staging system.

During peritoneal lavage, areas such as both sides of the diaphragm, beneath the liver, the omentum, the paracolic gutters, and the Douglas pouch should be flushed with saline. Avoiding flushing the primary lesion, and collect at least 100 mL of lavage fluid for examination.

Detection methods for FCCs in peritoneal lavage fluid include cytopathological examination and molecular biology techniques. Bando E, et al. tested FCCs in 1297 GC patients using cytology and found a 2% 5 year survival rate for CY1 cases, significantly lower than CY0 cases (*P* < 0.001) [26]. A meta-analysis of 26 studies involving 7970 patients confirmed that CY1 cases have significantly lower survival than CY0 cases (HR = 3.46, *P* < 0.0001) [27]. Cytopathological examination has high prognostic value but limited sensitivity and subjectivity, affecting accuracy. The detection rate of FCCs using cytology is about 5% in cases without visible PM and up to 30% in T4 stage cases [18, 26].

Molecular biology detection methods include transcription–reverse transcription concerted reaction (TRC), reverse transcription–polymerase chain reaction (RT–PCR), and reverse transcription loop-mediated isothermal amplification (RT–LAMP). A systematic review of 51 studies found that detecting carcinoembryonic antigen (CEA) messenger ribonucleic acid (mRNA) using RT–PCR is the most common molecular diagnostic method [18]. Although diagnostic value varies (sensitivity 38–100%, specificity 7.3–100%), molecular methods generally have higher sensitivity than traditional cytopathology.

Statement 4: Risk factors for GCPM include T3/T4 stage, lymph node metastasis (N +), extra-nodal invasion, large Borrmann type III or Borrmann type IV, diffuse type (Laurén classification), signet ring cell carcinoma, and tumor perforation or rupture.

(Quality of Evidence: B; Level of Recommendation: Strong; Degree of Expert Agreement: 96.4%).

The risk of GCPM is closely linked to tumor staging and pathological type. The main mechanism of PM is believed to originate from gastric serosal tumors. The frequency of PM notably increases once tumor cells infiltrate the serosa [28]. Studies indicate that 0.3% (3 of 1051) of patients with EGC and 3.2% (14 of 441) of patients with non-serosa-invasive GC experience PM after curative surgery [28, 29]. Higher T stage (particularly T4), extensive lymph node metastasis, Borrmann classification, and undifferentiated pathological types correlate with a higher incidence of PM [30].

Borrmann type III or IV GC present a risk of PM that is approximately 2.06 times higher than that of other types [30]. Large type III or type IV GC have a PM incidence ranging from 58.6% to 63.5% [31]. Diffused type GC according to the Laurén classification has a likelihood of PM exceeding 81.0% [32]. For T3 or T4 stage patients with lymph node involvement (N +), the incidence of PM is estimated at 25% [33]. Patients with N3a stage have a PM incidence 2.07 times greater than those with lymph node negativity, and N3b stage patients have a rate 3.44 times higher [30]. Additional high-risk factors for PM include extra-nodal metastasis, tumor perforation or rupture, signet ring cell carcinoma, mucinous adenocarcinoma, and poorly differentiated pathological types [34].

## Overview of treatment of PM from gastric cancer

Statement 5: Current treatments for GCPM include systemic chemotherapy, NIPS, HIPEC, and pressurized intraperitoneal aerosol chemotherapy (PIPAC).

(Quality of Evidence: C; Level of Recommendation: Weak; Degree of Expert Agreement: 92.9%).

Current guidelines suggest treating GCPM patients with systemic chemotherapy similar to other distant metastases, combining platinum, fluoropyrimidines and PD-1 antibody for first-line treatment [35–37]. However, due to limited drug penetration into the peritoneum from systemic administration, direct intraperitoneal chemotherapy is a reasonable approach. Common intraperitoneal treatments include NIPS, HIPEC, and PIPAC.

NIPS involves a chemotherapy port implanted in the abdominal wall, allowing intermittent delivery of chemotherapy at a consistent rate. This minimally invasive method provides prolonged drug action, easy administration, and strong sustainability [12], making it a primary therapeutic strategy for GCPM.

HIPEC, which is usually administered intraoperatively during surgery and may be repeated for a limited number of times, combines mechanical washing, thermally induced cytotoxic effects, and elevated local drug concentration [38], enhancing the cytotoxicity and permeability of chemotherapy drugs. For gastric cancer with a high risk of PM, the long-term outcomes of prophylactic HIPEC await the results of the HIPEC-01 study in China [39]. The efficacy of HIPEC combined with CRS for GCPM with low PCI scores is promising but still requires further validation [40–43]. The preliminary results of the HIPEC-02 study suggests that combining HIPEC with NIPS can be a potential treatment approach for GCPM [44].

The PIPAC uses pressurization to improve drug distribution and penetration within the abdominal cavity [45]. It combines the theoretical pharmacokinetic advantages of low-dose intraperitoneal chemotherapy (i.e., low toxicity, high intraperitoneal concentration, and low systemic concentration) with the principles of the aerosol (homogeneous intraperitoneal distribution and deeper tissue penetration) [46]. However, as an emerging technique, PIPAC's clinical application needs further exploration through extensive research.

Statement 6: NIPS offers significant benefits over systemic chemotherapy alone in treating GCPM.

(Quality of Evidence: B; Level of Recommendation: Weak; Degree of Expert Agreement: 96.4%).

The concept of NIPS was initially proposed in 2006[9], employing a tri-chemotherapeutic regimen on 96 patients with PM. This regimen included weekly intraperitoneal (IP) administration of docetaxel (40 mg) and carboplatin (150 mg) via a subcutaneously implanted IP port, combined with 100 mg/m^2^ of methotrexate and 600 mg/m^2^ of 5-fluorouraci via a peripheral vein. Before NIPS treatment, 63.9% of patients tested positive for FCCs within the abdominal cavity, which significantly declined to 27.9% post-treatment. Cytoreductive surgery (CRS) was performed on 30 patients, and the other 31 patients did not undergo surgery due to the progression of disease. The median survival time was 14.4 months, and the 1 year survival rates were 67%, indicating substantial efficacy improvement.

The PHOENIX studies, conducted by the University of Tokyo, provided an objective evaluation of NIPS in treating GCPM. The PHOENIX Phase III randomized controlled trial randomized 183 GCPM patients to either the IP group—treated with IP paclitaxel combined with systemic S-1/paclitaxel, or the SP group—treated with intravenous (IV) cisplatin and oral S-1[10]. The median overall survival (OS) for the IP and SP groups were 17.7 months and 15.2 months, respectively, with a Hazard Ratio (HR) of 0.72 and a 95% confidence interval (CI) of 0.49–1.04 (*P* = 0.08). The 3 year survival rates for the IP and SP groups were 21.9% and 6.0%, respectively. After adjusting for baseline ascites, the survival benefit for the IP group was significantly higher, with 17.7 months vs. 14.3 months for the SP group, with a HR of 0.64 and a 95% CI of 0.43–0.94 (*P* = 0.023). This advantage was especially evident in patients with moderate ascites, showing a more pronounced difference (13.0 months for the IP group vs. 6.8 months for the SP group), yielding a HR of 0.38.

Recent studies from Asian countries, including China, Japan, Korea, and Singapore, have suggested that IP administration of anticancer drugs is a viable method for treating GCPM [11, 47–54]. These studies reported conversion surgery rates of 41.9–78.0%, with R0 resection rates of 54.8–68.8%. Postoperative median survival time (MST) ranged from 12.8 to 43.2 months, and 3 year OS rates were between 12.0% and 55.3%. Chinese researchers further affirmed safety and efficacy of NIPS through the DRAGON-01 series of studies [11, 55–57].

## The basic theory of NIPS in the treatment of PM from gastric cancer

Statement 7: The plasma–peritoneal barrier limits the effectiveness of anti-tumor agents, especially larger molecules, in treating GCPM.

(Quality of Evidence: B; Level of Recommendation: Weak; Degree of Expert Agreement: 96.4%).

The principal advantage of IP therapy is the regional dose intensity. This process depends on the unique characteristics of the peritoneal–plasma barrier, which maintains a high concentration ratio of chemotherapeutic drugs between the peritoneal cavity and plasma. After IP administration, the drug must pass through the fluid in the abdominal cavity, the mesothelium, the interstitial space, and the blood vessel wall to reach the vascular compartment, forming the peritoneal–plasma barrier.

There are three exit routes from the peritoneum:

- Diffusion through the parietal peritoneal surfaces.
- Diffusion through the visceral peritoneal surfaces.
- Absorption through the lymphatic vessels.

Typically, water and solutes with a molecular weight less than 2000 are absorbed from the parietal and visceral peritoneum via the bloodstream. Lymphatic drainage on the diaphragmatic peritoneum, and to a lesser extent on the omentum and mesentery, serves as a significant mode of egress for particles the size of proteins, macromolecules, red blood cells, and tumor cells. The structure of the peritoneal–plasma barrier makes it difficult for larger molecular weight chemotherapy drugs to cross and affect PM foci during systemic treatment [58].

Statement 8: Intraperitoneal drug administration surpasses systemic administration in local drug concentration, duration of effectiveness and depth of infiltration.

(Quality of Evidence: B; Level of Recommendation: Weak; Degree of Expert Agreement: 85.7%).

The pharmacologic barrier between the peritoneal cavity and the plasma exhibits characteristics similar to the blood–brain barrier. In particular, large molecular weight drugs are cleared from the peritoneal cavity significantly slower than from the vascular compartment. Data suggest that the area under the curve (AUC, a measure of drug concentration over time) for IP drug delivery can be several orders of magnitude higher in the abdominal cavity compared to systemic drug exposure [59].

The drugs systemically administered rapidly distribute to various tissues, especially those with high blood flow, resulting in a rapid decrease in drug concentration in circulation and reducing the concentration gradient towards the peritoneal cavity. The relatively low blood supply of the peritoneum further slows down the transfer of drugs into the peritoneal cavity. Conversely, IP administration results in high drug concentrations within the peritoneal cavity and a substantial concentration gradient towards the circulation. After crossing the peritoneal–plasma barrier, drug molecules are rapidly transported to sites other than peritoneal tissue, maintaining a high concentration gradient over an extended period [58].

Pharmacokinetic studies confirm that IP administration results in a higher drug concentration and a prolonged duration of action compared to systemic administration. IP chemotherapy not only directly targets PM but also affects primary gastric tumors and metastatic lymph nodes [60]. Moreover, the absorption of medication through the portal vein may potentially mitigate liver metastasis. In addition, study using GFP-tagged human gastric cancer cell lines in nude mice have shown that intraperitoneal paclitaxel is significantly more effective against peritoneal micrometastasis compared to intravenous administration, confirming its potential in early phase treatment [61].

Statement 9: Slow absorption of the drugs from the peritoneal cavity does not exacerbate the systemic toxicity.

(Quality of Evidence: B; Level of Recommendation: Weak; Degree of Expert Agreement: 82.1%).

Due to the function of the plasma–peritoneal barrier, the absorption of intraperitoneally administered drugs, particularly paclitaxel, into the bloodstream is gradual, which helps minimize systemic toxic side effects. Ideally, drugs for IP chemotherapy should maintain a high concentration in the intraperitoneal fluid, be slowly absorbed into the bloodstream, and maintain a significant concentration gradient between the intraperitoneal fluid and plasma. These drugs should have a low clearance rate within the peritoneal cavity, be of large molecular weight, be water-soluble, easily dissolve, readily ionize, and have a high ratio of AUC for drug concentration over time in the peritoneal cavity compared to that in the peripheral blood. Thus, appropriate selection of drugs for intraperitoneal administration is of great importance.

In recent years, paclitaxel and docetaxel have received considerable attention for their role in IP chemotherapy. The ratio of the AUC of these two drugs in the peritoneal fluid to the AUC in plasma is 1000 and 550, respectively, underscoring their distinct pharmacokinetic advantage [62, 63]. Numerous clinical trials have demonstrated that the combination of paclitaxel, docetaxel, S-1, or oxaliplatin not only show promising clinical outcomes in the treatment of GCPM but also maintains a favorable safety profile [50, 64, 65]. Importantly, the PHOENIX–GC trial also demonstrated that IP paclitaxel was well-tolerated, with manageable non-hematologic toxicities comparable to systemic chemotherapy. Grade 3 or 4 adverse events related to IP ports were minimal and resolved with appropriate management, further supporting the safety profile of IP administration [10].

## Diagnostic methods and scoring of PM from gastric cancer

Statement 10: Enhanced abdominal CT is the preferred radiological method for detecting GCPM.

(Quality of Evidence: B; Level of Recommendation: Weak; Degree of Expert Agreement: 89.3%).

The diagnostic sensitivity of enhanced multidetector–row computed tomography (CT) for GCPM is approximately 50%, with specificity ranging from 95 to 99% [66, 67]. This accuracy surpasses that of ultrasound and PET–CT [68]. Multidetector–row CT can help determine the location and distribution of PM. Characteristic signs of PM include uneven thickening of the peritoneum, hyperintense or nodular presence, “omental cakes” or multiple omental bands and nodules, nodular thickening of the mesentery, and substantial ascites in the abdominopelvic cavity. Indirect signs include the dilation of the bile duct, ureter, and intestine.

With the rapid development of computer technology, radiomics and artificial intelligence (AI) technologies have significantly improved diagnostic accuracy, therapeutic response evaluation, and prognostic assessment for GCPM. Radiomics can extract features from large volumes of medical images that are difficult to discern with the naked eye, allowing for a more comprehensive and objective assessment of lesion characteristics [69, 70].

The diagnostic efficacy of PET–CT depends on the uptake rate of FDG (18F-fluorodeoxyglucose) by cancer cells, which correlates with the expression of glucose transporter 1 (GLUT1). GLUT1 expression was varied in different histological types of gastric cancer. The most common histological types of PM, such as signet ring cell carcinoma, mucinous adenocarcinoma, or poorly differentiated adenocarcinoma, exhibit extremely low rates of GLUT1 expression [71]. The use of FAPI (Fibroblast Activation Protein Inhibitor), a new metabolic imaging agent for PET–CT, can potentially enhance the diagnostic efficacy of GCPM [72–74].

Magnetic resonance imaging (MRI) can be used as an alternative to enhanced CT scanning which are contraindicated for some patients. Although MRI is less accessible and more complex to interpret than CT, studies have reported that diffusion-weighted MRI (DW-MRI) sometimes outperforms CT scans in detecting PM. For instance, for right diaphragm PM, the AUC for DW-MRI was 0.95 compared to 0.81 for CT; for left diaphragm PM, the AUC was 0.89 for DW-MRI compared to 0.74 for CT. For omental PM, liver surface, bowel mesentery, and cul de sac, the AUCs were similar or slightly better for DW-MRI compared to CT [75].

Statement 11: Diagnostic laparoscopy with peritoneal cytology and nodule biopsy is the most reliable method for diagnosing and assessing suspected GCPM.

(Quality of Evidence: A; Level of Recommendation: Strong; Degree of Expert Agreement: 100%).

As previously stated, the sensitivity of imaging techniques in identifying PM is notably limited. Currently, the most reliable method for diagnosing suspected PM and assessing its extent combines diagnostic laparoscopy, peritoneal cytology, and peritoneal nodule biopsy. The National Comprehensive Cancer Network (NCCN) guidelines recommend staging laparoscopy and the detection of FCCs before treatment for GC patients with a stage of T3 or N1 and above [37]. Cytology testing on peritoneal fluid significantly refines the staging laparoscopy process by detecting occult carcinomatosis [76]. The results from diagnostic laparoscopy, combined with peritoneal cytology and peritoneal nodule biopsy, are crucial for formulating clinical treatment strategies [77–80].

A prospective study in Japan, involving 156 asymptomatic patients, highlighted the importance of diagnostic laparoscopy and cytological detection [81]. The study included patients with: (1) large Borrmann type 3 tumors measuring ≥ 8 cm, (2) Borrmann type 4 tumors (known as linitis plastica), (3) swollen bulky lymph nodes or para-aortic lymph node, or (4) a clinical suspicion of peritoneal disease. As a result of diagnostic laparoscopy and cytological detection, the initial treatment strategy had to be altered for 47% of the patient due to the newly discovered presence of occult PM.

Statement 12: PM is classified into P0CY1, P1a, P1b, and P1c categories, using the peritoneal cancer index (PCI) to assess the extent and location of metastasis.

(Quality of Evidence: A; Level of Recommendation: Weak; Degree of Expert Agreement: 82.1%).

Comprehensive categorization aids in the implementation of more precise therapeutic strategies and improves prognostic stratification. According to the classification by JGCA:

- PX: Inability to determine the presence of PM.
- P0: Absence of PM.
- P1: Presence of PM, which is further divided into P1a, P1b, and P1c.
- P1a: Localized PM (confined exclusively to the peritoneum surrounding the stomach, greater omentum, lesser omentum, anterior leaf of the transverse colon, pancreatic capsule, spleen, etc.).
- P1b: Metastasis to the upper abdomen.
- P1c: Metastasis to the mid-lower abdomen.

Positive peritoneal cytology is linked to a poor prognosis in GC patients and is an independent predictor for recurrence following curative resection [82]. Therefore, positive peritoneal cytology even in the absence of visible peritoneal implants (P0CY1) should be considered a distinctive form of PM.

**Fig. 1:**
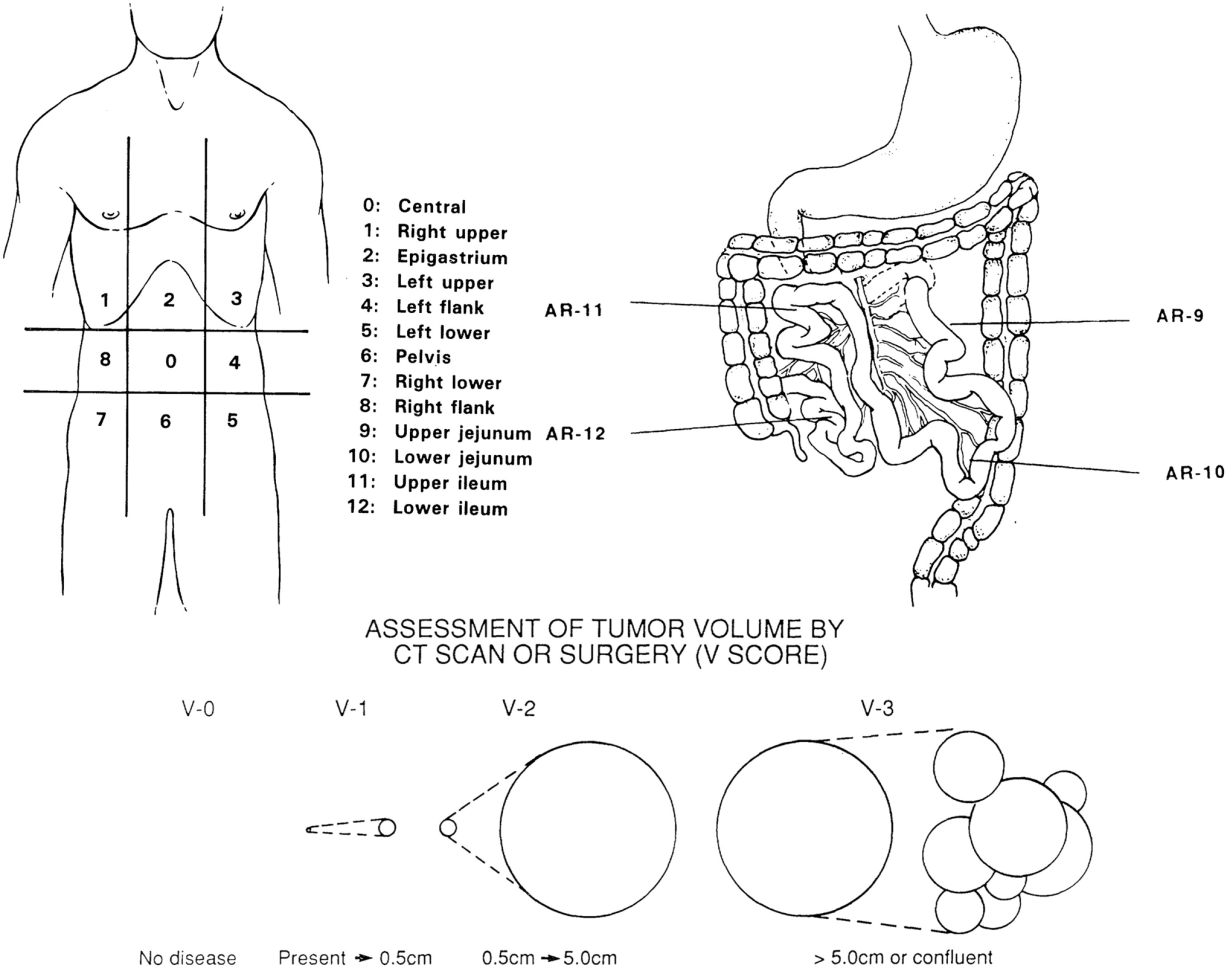
Illustration of the peritoneal cancer index (PCI) scoring system [83]

As early as 1996, Paul H. Sugarbaker proposed the peritoneal cancer index (PCI) as a method to evaluate the extent of PM [83]. This scoring system segments the abdominal cavity into nine regions and the small intestine into an additional four regions, yielding a total of thirteen regions for assessment. Each region is assigned a score ranging from 0 to 3, reflecting the dimensions of the lesion observed during surgical procedures. This scoring system has gained widespread acceptance for evaluating PM. During diagnostic laparoscopy, the lesion size (LS) in each of the thirteen regions is determined and scored, yielding a maximum possible score of 39. This cumulative score is then used to assess the dispersion of PM. However, the practical implementation of the PCI evaluation can prove challenging, and the precision and feasibility of its application necessitate further investigation (Fig. 1).

## The indications for laparoscopic exploration

Statement 13: Patients with cT3/T4 or high PM risk should undergo diagnostic laparoscopy and FCC examination.

(Quality of Evidence: A; Level of Recommendation: Weak; Degree of Expert Agreement: 89.3%).

A population-based study involving 5220 GC patients found 706 cases of PM (14% of the total) [7]. Factors such as younger age (less than 60 years), female gender, advanced T- and N-stage, primary tumor of signet ring cells or linitis plastica, and multiple stomach tumors were all associated with higher odds ratios of developing PM. Potential risk for GCPM include cT3/T4, N  + , extra-nodal invasion, Borrmann type 3 or type 4, diffuse type as defined by Laurén's classification, signet ring cell carcinoma, and the presence of tumor perforation or rupture.

The PLASTIC study, compared staging laparoscopy with PET–CT, highlights the value of staging laparoscopy in detecting PM, changing treatment intent from curative to palliative in 16% of locally advanced gastric cancer patients [84].

For patients being evaluated for surgical resection, whether or not they have undergone preoperative treatment, staging laparoscopy combined with an examination for FCCs may prove beneficial. This approach can help identify PM that radiological imaging fails to detect, particularly in individuals who exhibit the aforementioned risk factors. However, for cT3 cases, the decision to perform diagnostic laparoscopy can be based on the availability of this procedure.

Statement 14: For GCPM patients treated with preoperative NIPS, a second-look laparoscopy is recommended to evaluate the treatment response and potential suitability for surgery, provided the treatment is effective and the patient is in good physical status.

(Quality of Evidence: D; Level of Recommendation: Weak; Degree of Expert Agreement: 96.4%).

Typically, the response to chemotherapy is assessed after every 2–3 treatment cycles, corresponding to roughly every 2 months, although the duration varies depending on the severity of the initial peritoneal dissemination. This evaluation includes the examination of the patient's general condition, tumor markers, volume of ascites, and changes in the primary lesion and PM [56]. For patients identified as potentially eligible for surgery through radiological evaluation, a secondary laparoscopic exploration is recommended to confirm their suitability for conversion surgery. If a patient does not satisfy the criteria for surgery, treatment will be extended for an additional 2–3 cycles, after which the patient will be reevaluated.

Considering the invasiveness of laparoscopic exploration, it is advisable to perform such a procedure after every 3–6 treatment cycles. If the objective of the laparoscopic exploration is to assess the indications for conversion surgery, then the necessary conditions should include clear evidence of treatment efficacy and the patient maintaining an overall satisfactory performance status.

## Appropriate subjects and indications for NIPS

Statement 15: NIPS is recommended for patients with confirmed PM (P1CY0/1) or positive FCC findings (P0/1CY1) from laparoscopy.

(Quality of Evidence: B; Level of Recommendation: Weak; Degree of Expert Agreement: 85.7%).

Following the successful outcomes of phase II studies [85, 86] demonstrating the efficacy and safety of IP paclitaxel, the first randomized controlled trial (RCT) is reported in 2018 [10]. This trial involved 183 patients with GCPM, randomly allocated in a 2:1 ratio to either the IP group or the SP group. The IP group received IP paclitaxel (20 mg/m^2^ on days 1, 8), IV paclitaxel (50 mg/m^2^ on days 1, 8), and oral S-1 (80 mg/m^2^ on days 1–14) in a 3-week cycle. The SP group received IV cisplatin (60 mg/m^2^ on day 8) and oral S-1 (80 mg/m^2^ on days 1–21) in a 3 cycle.

The results revealed that NIPS treatment with IP paclitaxel significantly increased the rate of successful conversion to FCCs (76% vs. 33%). Three-year follow-up results indicated a higher survival rate in the IP group at 21.9% compared to 6.0% in the SP group. After adjusting for baseline ascites, the survival benefit for the IP group was significantly more pronounced (Hazard ratio was 0.59, 0.39 to 0.87; *P* = 0.008). These findings suggested that NIPS treatment could be recommended as a treatment option for GCPM. Extensive research undertaken by Chinese teams on NIPS treatment aims to further validate and corroborate its safety and efficacy [11, 55–57].

Statement 16: NIPS treatment is not recommended for patients with severe abdominal adhesions, PM-induced intestinal obstruction, or other life-threatening metastases.

(Quality of Evidence: C; Level of Recommendation: Weak; Degree of Expert Agreement: 82.1%).

Due to the requirement of laparoscopic exploration both before and during NIPS to establish the IP chemotherapy port and assess the treatment efficacy, the contraindications for NIPS also apply to laparoscopic exploration. Patients with a history of extensive abdominal and pelvic surgery, suspected severe abdominal adhesions, or insufficient cardiopulmonary function to tolerate anesthesia and carbon dioxide pneumoperitoneum are not suitable candidates for laparoscopic surgery [87].

According to the principle of NIPS treatment, the chemotherapy solution must be evenly distributed throughout the abdominal cavity to maximize its effectiveness. Severe abdominal adhesions can obstruct laparoscopic procedures and hinder the distribution of the chemotherapy solution, making NIPS treatment unsuitable.

In cases where PM leads to intestinal obstruction, the tumor burden is obviously high, indicating a terminal stage, where the patient is unlikely to tolerate chemotherapy. Due to the numerous uncertainties in treating malignant intestinal obstructions and other life-threatening distant metastasis, RCTs are challenging to conduct, resulting in relatively low-quality evidence [88]. Given that the expected survival time for these patients is generally short and clinical management mainly focus on best supportive care (BSC), NIPS is not recommended.

## The regimen for intraperitoneal treatment

Statement 17: Paclitaxel is the common first-line IP chemotherapy agent for NIPS, with strongest level of evidence support for 20 mg/m^2^ on days 1, 8 every 3 weeks.

(Quality of Evidence: C; Level of Recommendation: Weak; Degree of Expert Agreement: 92.9%).

IP therapy involves introducing chemotherapeutic agents directly into the abdominal cavity, bypassing the blood–peritoneal barrier. This approach allows for direct interaction between the drug and the tumor, enhancing its anticancer effects. The criteria for selecting drugs for IP administration include sensitivity to primary tumor, high penetration, substantial molecular weight, low peritoneal absorption, and minimal peritoneal irritation [89]. Paclitaxel, with its substantial molecular weight and lipophilic properties, is slowly absorbed via the lymphatic system and exhibits strong antiproliferative activity, thus rarely causing intra-abdominal adhesions. This property permits repeated administrations within the abdominal cavity, making paclitaxel an ideal agent for IP chemotherapy [64, 90].

The strongest evidence for NIPS treatment comes from a phase III RCT that uses a regimen of IP paclitaxel at a dosage of 20 mg/m^2^ on days 1 and 8 every 3 weeks. Based on the outcomes of this trial, this regimen is recommended as the first-line therapy for IP treatment [10]. However, several small-sample phase I and II clinical trials have also explored higher doses of IP paclitaxel (40–80 mg/m^2^), demonstrating promising efficacy and safety profiles [8, 86, 91, 92]. These trials indicate that higher doses can be well-tolerated and may offer beneficial therapeutic outcomes, although more research is needed to confirm their efficacy compared to the standard dose.

Statement 18: Common IP chemotherapy agents for NIPS include paclitaxel, docetaxel, and cisplatin, with paclitaxel being preferred for its higher lipophilicity.

(Quality of Evidence: B; Level of Recommendation: Weak; Degree of Expert Agreement: 85.7%).

Commonly used IP chemotherapy drugs include paclitaxel, docetaxel, and cisplatin[16]. The dosage, varieties, and frequencies of IP drugs applied in various studies lack uniformity. Paclitaxel is the most frequently administered drug in NIPS treatment, with reported dosages ranging from 20 mg/m^2^ to 80 mg/m^2^ (Table 1). However, there is no evidence indicating that higher dosages of paclitaxel lead to improved therapeutic effectiveness.

**Table 1:** Regimens of drugs commonly used for IP treatment

IP treatment	Systemic treatment
PTX 20 mg/m^2^ days 1, 8	PTX 50 mg/m^2^ IV days 1, 8 + S-1 40 mg/m^2^ BID PO days 1–14 (PS regimen) [10, 85]
PTX 20 mg/m^2^ days 1, 8, 22	CDDP 60 mg/m^2^ IV day 8 + S-1 40 mg/m^2^ BID PO days 1–14 (SP regimen) [52]
PTX 40–80 mg/m^2^ days 1, 8	OXA 100 mg/m^2^ IV day 1 + S-1 40 mg/m^2^ BID PO days 1–14 (SOX regimen) [65]
PTX 40 mg/m^2^ days 1, 8	OXA 100 mg/m^2^ IV day 1 + CAPE 1000 mg/m^2^ BID PO days 1–14 (CAPEOX regimen) [96]
PTX 60 mg/m^2^ day 1	OXA 100 mg/m^2^ IV day 1 + LV 100 mg/m^2^ IV day 1 + 5-FU 100 mg/m^2^ IV day 1 (FOLFOX regimen) [54]
PTX 80 mg/m^2^ day 1	OXA 100 mg/m^2^ IV day 1 + S-1 40 mg/m^2^ BID PO days 1–14 (SOX regimen) [97]
PTX 80 mg/m^2^ days 1, 8	PTX 50 mg/m^2^ IV days 1, 8 + S-1 40 mg/m^2^ BID PO days 1–14 (PS regimen) [92]
DOC 40–60 mg/m^2^ days 1, 8	S-1 40 mg/m^2^ BID PO days 1–14 [93]
DOC 35–50 mg/m^2^ days 1, 15	S-1 40 mg/m^2^ BID PO days 1–14 [98]
CDDP 30 mg/m^2^ day 1 + DOC 30 mg/m^2^ day 1	S-1 30 mg/m^2^ BID PO days 1–14 [99]

In a dose-escalation trial using IP PTX + SOX regimen, S-1 was orally administered twice daily at 80 mg/m^2^/d for 14 days, followed by a 7-day rest. On day 1, oxaliplatin 100 mg/m^2^ was given intravenously, and IP PTX was given on days 1 and 8[65]. The initial dose of IP PTX was 40 mg/m^2^, with dose increments of 20–80 mg/m^2^. Dose-limiting toxicity (DLT) was defined as grade 3 non-hematological toxicity, grade 4 leukopenia, grade 3 febrile neutropenia, and grade 3 thrombocytopenia. The results determined that the dose of IP PTX in combination with systemic SOX was 80 mg/m^2^.

A recent phase II study also explored the efficacy of IP PTX combined with S-1 and cisplatin for GCPM [52]. Patients received IP PTX on days 1, 8, and 22 at a dose of 20 mg/m^2^, in addition to an established S-1 and cisplatin regimen every 5 weeks. The OS at 1 year was 73.6% and the median survival time was 19.4 months. Notably, this regimen resulted in less chemotherapy-induced neurotoxicity and better tolerability compared to the IP PTX + SOX regimen.

Another study evaluates the efficacy and safety of IP docetaxel combined with oral S-1 for GCPM [93]. Eighteen patients underwent two cycles of NIPS followed by gastrectomy if no macroscopic PM remained. Results showed that 78% of patients had negative peritoneal cytology and no macroscopic PM after NIPS, with a median survival time of 24.6 months and minimal severe toxicities, suggesting that IP use of docetaxel is also a viable option for NIPS.

A study indicates that cisplatin, with its low molecular weight and water-soluble properties, is rapidly absorbed by the peritoneal mesothelium, thus not offering significant pharmacokinetic benefits in IP administration [94]. However, the combined use of paclitaxel and cisplatin may be effective against paclitaxel-resistant metastatic lesions. In addition, several drugs including mitomycin C, oxaliplatin, doxorubicin, etoposide, and irinotecan have been documented as safely administered in HIPEC treatment. Thus, they could potentially be considered for NIPS treatment [16, 95].

Consolidating the published literature, the regimens of drugs commonly used for IP applications are presented in Table 1.

Statement 19: Chemotherapy agents for IP therapy are usually dissolved in about 500–1000 mL of perfusate for patients without extensive ascites.

(Quality of Evidence: D; Level of Recommendation: Strong; Degree of Expert Agreement: 100%).

The choice of solvent volume for dissolving chemotherapeutic agents is crucial for their effective distribution in the peritoneal cavity. The optimal carrier solution for IP chemotherapy should ensure prolonged exposure of all tumor lesions to high concentrations of cytotoxic agents and a homogeneous distribution of the drug [100]. Insufficient volumes of fluid do not circulate freely within the peritoneum, even with frequent alterations in the patient's position [101]. Conversely, larger volumes (> 2 L/m^2^ body surface area) that facilitate a more homogenous IP drug distribution may induce abdominal distension.

Currently, most research uses 1000 mL of normal saline as a solvent for IP chemotherapy for patients without extensive ascites, providing beneficial therapeutic outcomes and patient comfort with reduced risk of abdominal distension [16, 95]. Studies also indicate that using 500 mL of perfusate can be effective in IP chemotherapy [102]. In addition, literature suggests that for patients with a body surface area (BSA) > 2.0 m^2^, the volume of intraperitoneal infusion can be increased to 1500 mL (or 700 mL/m^2^) [103]. Therefore, it is recommended to use this dosage for NIPS treatment. For patients with moderate to large amounts of ascites, it may be considered to drain some ascitic fluid via a port before NIPS treatment or to reduce the volume of intraperitoneal injection accordingly.

## Prevention and treatment of complications after placement of intraperitoneal chemotherapy port

Statement 20: Complications of IP port implantation include port-related infection, inadequate fixation, subcutaneous fluid accumulation, incision infection or dehiscence, inflow obstruction, catheter damage or rupture, subcutaneous tumor metastasis, etc.

(Quality of Evidence: C; Level of Recommendation: Strong; Degree of Expert Agreement: 96.4%).

Currently, an IP port and catheter system is the main method for administering NIPS treatment due to its lower complication rate compared to a single-use catheter [104]. Complications related to IP port implantation include infection around the port, inadequate fixation, extravasation or reflux of chemotherapeutic agents, infection or dehiscence of the incision, port obstruction, catheter damage or rupture, and the potential risk of subcutaneous tumor metastasis [105, 106].

Port-related infection is identified if the port-site skin shows redness, tenderness, fluctuation, or purulence, with or without fever, elevated white blood cell (WBC) count, or increased C-reactive protein (CRP) level. Inadequate fixation is characterized by displacement of the port, rotation, or angulation with the skin, leading to difficulties with needle insertion. Inflow obstruction is defined as the inability to infuse saline into the abdominal cavity via the catheter. Subcutaneous fluid accumulation could be result from the reflux of fluid from the abdominal cavity along the catheter or from inflammatory exudate.

To assess the severity of port-related complications, Yang et al. proposed a classification method based on the Clavien–Dindo classification [105]:

- Grade 1: Minor subcutaneous fluid accumulation or slight rotation, with the port remaining functional without the necessity for pharmacological treatment or surgical interventions.
- Grade 2: Mild subcutaneous fluid accumulation, minor wound dehiscence, mild infection, or rotation, manageable by conservative treatments such as pharmacological interventions, without impeding IP chemotherapy.
- Grade 3: Moderate subcutaneous fluid accumulation, wound dehiscence, infection, or rotation necessitating surgical intervention before the port could be reused.
- Grade 4: Severe port complications requiring immediate intervention, rendering the port unusable and necessitating its removal or replacement.

Statement 21: Measures to prevent IP port complications include: proper fixation of the port and secure embedding of the catheter.

(Quality of Evidence: D; Level of Recommendation: Weak; Degree of Expert Agreement: 100%).

Retrospective analysis indicates a significant association between port-related complications and factors, such as the Eastern Cooperative Oncology Group (ECOG) performance status, serum albumin levels, the implantation procedure, and the experience of the implantation group [105]. The standard protocol for IP port implantation should include secure fixation of the port, immobilization and embedding of the catheter and thorough irrigation of the incision site. To optimize the procedure, the catheter's entrance into the aponeurosis should be secured, and the exposed portion of the catheter should be embedded using nonabsorbable sutures [105].

The primary causes of inflow obstruction are intraperitoneal adhesions and sheath formation around the catheter. Therefore, the catheter’s length should not be excessively long to avoid catheter obstruction. Although some researchers suggest a correlation between infection and concurrent gastrointestinal surgery with port implantation, retrospective analyses have not found empirical evidence supporting this correlation [107]. If gastrointestinal surgery and IP port implantation must be performed concurrently, it is recommended to proceed with the implantation of the IP port before disconnecting the gastrointestinal tract.

To mitigate the risk of port-related infections, medical staffs are advised to adhere to stringent aseptic principle, including thorough hand washing, wearing gloves, meticulous disinfection of the port area, wearing masks, and maintaining silent prior to needling. In addition, adhering to the principles of no-touch techniques during implantation and ensuring a tumor-free environment are crucial to prevent tumor cell dissemination and infection.

## The indications for conversion surgery

Statement 22: Criteria for conversion surgery include but not limited to obvious shrinkage of PM on second laparoscopy, negative peritoneal cytology, no other distant metastases, resectable primary tumor, and good performance status of the patient.

(Quality of Evidence: C; Level of Recommendation: Strong; Degree of Expert Agreement: 100%).

Based on the classification of metastatic GC proposed by Kazuhiro Yoshida, conversion surgery is considered rational for patients who show a favorable response to treatment, complete regression of PM, and negative peritoneal cytology [108]. However, due to the challenges in differentiating between residual tumor and fibrosis or scar tissue, conversion surgery for GCPM is considered viable when there is a significant shrinkage of PM observed during the second laparoscopy. This perspective is widely supported by numerous studies [11, 55, 56, 109–111]. Building upon this foundation, more explicit criteria for conversion surgery following NIPS treatment are proposed [56]:

- Obvious shrinkage of PM as observed in a second laparoscopy.
- Negative results in peritoneal cytology.
- Absence of other distant metastases.
- Downstaging of the primary tumor to a resectable state.
- Good performance status of the patient.

Even if patients with PM successfully undergo conversion surgery, the proportion that achieves complete remission and sustains long-term survival remains low. In a phase II clinical trial in Japan, among 100 patients, 64 underwent conversion surgery and achieved a median survival duration of 30.5 months. However, despite the continuation of IP chemotherapy postoperatively, 58 patients (91%) experienced disease recurrence and progression, with 66% of these recurrences still manifesting as PM [109]. A phase II clinical trial in China showed similar results. Patients who underwent conversion surgery and achieved R0 resection had a median survival duration of 31.3 months, while those who underwent R2 resection had a significantly lower median survival of 15.8 months [11].

Achieving complete eradication of the disease via surgical intervention in patients with GCPM is exceptionally challenging. Strict control of surgical indications to prevent R2 resection, coupled with sustained postoperative treatment and intensive patient monitoring, are critical for improving prognosis.

## Recommended surgical procedures

Statement 23: If the criteria for conversion surgery are fulfilled, it is recommended to proceed with gastrectomy with R0 intent.

(Quality of Evidence: C; Level of Recommendation: Strong; Degree of Expert Agreement: 100%).

Retrospective analyses indicate that patients undergoing R0 resection have significantly better prognoses compared to those with R1 or R2 resections [110, 112]. When conversion surgery criteria are met, a gastrectomy with R0 intent is recommended. Recent studies support the feasibility of laparoscopic surgery for stage IV gastric cancer, including cases with peritoneal metastasis [113, 114]. However, if the primary lesion directly invades surrounding organs (T4b) or involves bulky metastatic lymph nodes (Bulky N2), the complexity of laparoscopic procedures increases, making open surgery a more viable option. If it can be safely executed, D2 lymphadenectomy is recommended to achieve radical resection of the primary lesion. For patients presenting with a substantial tumor burden and deemed incurable but show a good response to treatment, cytoreductive surgery might be considered after multidisciplinary deliberation.

## The protocol and duration of intraperitoneal therapy after conversion surgery

Statement 24: Start intraperitoneal adjuvant chemotherapy within 4–6 weeks after conversion surgery, using the same regimen as before.

(Quality of Evidence: C; Level of Recommendation: Weak; Degree of Expert Agreement: 96.4%).

For patients with PM, achieving a genuine cure through surgical intervention is extraordinary challenging, necessitating sustained treatment following surgery. Most literature on perioperative treatment suggests that adjuvant chemotherapy should start within 4–6 week post-surgery [31, 56, 115, 116]. This interval balances the surgical safety with the urgency of cancer treatment, supported by phase III RCTs to affirm its feasibility. Retrospective studies have indicated that early postoperative intraperitoneal chemotherapy (EPIC) could enhance overall survival and GC-specific survival [117]. Therefore, providing surgical safety is assured, early application of IP chemotherapy can be attempted to achieve tumor resistance and prevent surgical-induced abdominal adhesions.

Statement 25: Maintain IP chemotherapy until disease progression or unmanageable toxicity occurs after conversion surgery.

(Quality of Evidence: C; Level of Recommendation: Weak; Degree of Expert Agreement: 92.9%).

While NIPS treatment may induce port-related complications, the slow absorption of drugs from the peritoneal cavity into the bloodstream following IP administration does not increase systemic toxic side effects, allowing for prolonged NIPS treatment. Most clinical trial protocols for NIPS treatment stipulate that patients should adhere to the prescribed regimen until disease progression, intolerable toxicity, the investigator's decision, or patient withdrawal occurs [10, 95, 118]. Based on this, the consensus is to continue IP chemotherapy until there is demonstrable disease progression or unmanageable toxic reactions after conversion surgery. In addition, an empirically based suggestion proposes continuing NIPS treatment for a minimum of 3 years, as most patients likely experience PM progression within this timeframe. If the disease remains stable beyond 3 years, the mortality risk associated with PM appears to decrease.

## Management of GCPM patients with ovarian metastases

Statement 26: For untreated GCPM patients with asymptomatic ovarian metastasis without significant ascites, NIPS treatment to be recommended instead of direct oophorectomy.

(Quality of Evidence: C; Level of Recommendation: Weak; Degree of Expert Agreement: 85.7%).

For untreated GCPM patients with asymptomatic ovarian metastasis and no significant ascites, NIPS treatment is recommended over direct oophorectomy. This approach aligns with evolving understanding that ovarian metastasis in gastric cancer is driven by multiple mechanisms, including lymphatic and hematogenous spread, alongside implantation metastasis [119]. Previously, peritoneal implantation metastasis was considered the primary mode of transmission; however, the smooth and intact surface of ovarian metastatic tumors and instances, where GC is confined to the mucosal and submucosal layers without PM challenge this theory [120]. Currently, it is postulated that ovarian metastasis is the result of several metastatic mechanisms acting together.

Prognostic factors for ovarian metastasis include the primary tumor's clinicopathologic features, presence and extent of PM, ovarian lesion size, intraperitoneal fluid volume, and additional organ metastases [121–123]. Typically, for patients with ovarian metastasis and no other distant metastasis, if the primary tumor is resectable and does not require preoperative treatment, simultaneous resection of the ovarian metastasis is recommended. However, the rates of PM from synchronous and metachronous ovarian metastasis in GC are reported to be 83.2% and 78%, respectively [124].

When ovarian metastasis is accompanied by PM but lacks significant symptoms or ascites, initial NIPS treatment is recommended. This can help in controlling PM, allowing for subsequent combined gastrectomy and oophorectomy. If ovarian metastasis is not accompanied by macroscopically visible PM and the primary tumor requires preoperative treatment, prophylactic NIPS treatment can be considered to mitigate the risk of peritoneal metastasis and recurrence.

Statement 27: For symptomatic ovarian metastasis, oophorectomy is first recommended, followed by NIPS treatment.

(Quality of Evidence: C; Level of Recommendation: Weak; Degree of Expert Agreement: 92.9%).

For symptomatic ovarian metastasis, an oophorectomy is recommended as the initial step, followed by NIPS treatment. This approach is supported by evidence indicating that an early oophorectomy can reduce symptoms caused by large ovarian tumors and ascites, thus improving the patient's quality of life and preparing them for subsequent treatments [121].

In cases where ovarian metastatic tumors are large and symptomatic but not adherent or fixed, bilateral ovariectomy can be considered prior to initiating systemic therapy or NIPS. This surgical intervention aims to not only ease symptoms but also potentially confer survival benefits if the metastasis shows resistance to chemotherapy.

In young patients with unilateral ovarian metastasis, preservation of the normal contralateral ovary may be an option if there is no evidence of metastasis and upon obtaining informed consent. Otherwise, a prophylactic contralateral ovariectomy is advised to prevent potential future metastasis [122].

## NIPS treatment for postoperative peritoneal recurrence or metastasis

Statement 28: For postoperative PM, attempt laparoscopic exploration. If adhesion is not severe, consider NIPS treatment.

(Quality of Evidence: C; Level of Recommendation: Weak; Degree of Expert Agreement: 89.3%).

When patients experience peritoneal recurrence or metastasis postoperatively, treatment becomes exceedingly arduous, and outcomes are often frustrated. Recent clinical trials, such as PHOENIX–GC, have demonstrated the safety and potential of NIPS treatment for synchronous GCPM [10]. However, there have been limited investigations into the efficacy of NIPS treatment for metachronous PM following curative surgery for GC. As proved by the findings of the ACTS–GC, CLASSIC, and JACCRO GC-07 trials, the standard protocol for patients with peritoneal recurrence following gastrectomy is systemic chemotherapy [125–127].

Considering that NIPS treatment has shown minimal systemic toxicity and offers efficient and intensive regional therapy compared with systemic chemotherapy, it is advisable for patients experiencing peritoneal recurrence postoperatively to consider laparoscopic exploration. If the peritoneal adhesion is not severe, NIPS treatment remains a viable option [128].

## Systemic treatment in NIPS treatment

Statement 29: Systemic treatment in NIPS treatment follows the guidelines for metastatic GC, including chemotherapy, targeted therapy and immunotherapy.

(Quality of Evidence: A; Level of Recommendation: Weak; Degree of Expert Agreement: 89.3%).

Systemic chemotherapy is an effective treatment for metastatic GC, superior to best supportive care. PM is a local manifestation of GC, and systemic treatment is the standard treatment for GCPM. Although the highest level of evidence for NIPS therapy is the combination of S-1 with IV and IP PTX, the increasing evidence supporting various systemic chemotherapy regimens, targeted therapies, and immunotherapies for metastatic GC suggests that these can also incorporated in NIPS treatment.

Commonly used systemic chemotherapy regimens in NIPS are typically two-drug regimens, which are more effective than single-drug regimens and less toxic than three-drug regimens. Common regimens include XELOX (Capecitabine + Oxaliplatin) [129], SOX (S-1 + Oxaliplatin) [130], SP (S-1 + Cisplatin) [52], PS (Paclitaxel + S-1) [10], etc. Immune checkpoint inhibitors such as nivolumab [131] and pembrolizumab [132] can be added, especially for patients with high combined positive score (CPS). For HER2 (+) patients, trastuzumab can be included [133]. For patients positive for CLDN18.2, Zolbetuximab can be combined with chemotherapy, as demonstrated in the SPOTLIGHT [134] and GLOW studies [135].
